# The establishment of a growth-controllable orthotopic bladder cancer model through the down-regulation of c-myc expression

**DOI:** 10.18632/oncotarget.10784

**Published:** 2016-07-22

**Authors:** Ho Kyung Seo, Seung-Phil Shin, Na-Rae Jung, Whi-An Kwon, Kyung-Chae Jeong, Sang-Jin Lee

**Affiliations:** ^1^ Center for Prostate Cancer, Hospital, National Cancer Center, Goyang, Gyeonggi-do, Korea; ^2^ Genitourinary Cancer Branch, Research Institute, National Cancer Center, Goyang, Gyeonggi-do, Korea; ^3^ Department of Urology, School of Medicine, Institute of Wonkwang Medical Science, Wonkwang University, Wonkwang University Sanbon Hospital, Gunpo, Gyeonggi-do, Korea; ^4^ Biomolecular Function Research Branch, Research Institute, National Cancer Center, Goyang, Gyeonggi-do, Korea

**Keywords:** bladder cancer, c-myc, orthotopic bladder cancer model, MBT-2

## Abstract

To properly evaluate the biological effects of immunotherapy, it is critical to utilize a model of cancer in immune-competent mice. Currently, MBT-2 is the most common murine bladder cancer cell line used in orthotopic bladder cancer models, even though this cell type often has an inappropriate genetic mutation landscape. In these models, after tumors are detected with *in vivo* imaging, the mouse usually dies within two to three weeks due to post-renal azotemia caused by the rapidly growing mass. This event prohibits the evaluation of tumor behavior upon intravesical drug treatment. We explored whether an shRNA-induced decrease in the expression of the *c-myc* oncogene in MBT-2 cells could slow down their *in vitro* proliferation and *in vivo* tumor growth. We transduced MBT-2 cells with shRNA lentiviruses that bound c-myc, established MBT2.cMYCshRNA and confirmed the retardation of the growth of tumors implanted in C3H/He mice. Accordingly, this study suggests that this novel orthotopic bladder cancer model in immune-competent mice may be more appropriate for the analysis of the effects of the intravesical instillation of immunotherapeutic agents.

## INTRODUCTION

Non-muscle-invasive bladder cancer (NMIBC) accounts for > 70% of all newly diagnosed cases of bladder cancer. The gold standard for the treatment of patients with NMIBC is transurethral resection. Major obstacles in the treatment of this cancer include the high incidence of tumor recurrence and the progression to muscle-invasive disease. To prevent recurrence and progression, intravesical Bacillus Calmette-Guerin (BCG) immunotherapy or intravesical chemotherapy are used as adjuvant therapies; however, a substantial number of patients do not complete this therapy owing to refractory disease or intolerance [1]. Novel intravesical treatment strategies with improved efficacy and lower toxicity are urgently needed. Immunotherapy holds promise in the battle against late-stage and metastatic bladder cancer, which have a poor prognosis [2]. The discovery of immune checkpoint proteins, such as CTLA-4 (cytotoxic T-lymphocyte-associated protein 4), PD-1 (programmed death-1), TIM-3 (T-cell immunoglobulin and mucin-domain containing-3), LAG-3 (Lymphocyte-activation gene 3), and IDO (Indoleamine 2,3-dioxygenase), along with a better understanding of the role that they play in the mechanisms of immune evasion in tumors have facilitated the production of a significant anti-tumor immune response in patients with cancer. For example, the use of monoclonal antibodies to block the inhibitory signals of the programmed death-1 (PD-1)/programmed death-ligand 1 (PD-L1) pathway has demonstrated potential for chemotherapy-resistant patients [2]. A number of other cancer immunotherapies are under investigation, but few immune-competent mouse models for bladder cancer exist.

To evaluate the effects of new immunotherapeutic modalities, it is essential to use a suitable immune-competent animal model that mimics the behavior of human disease. Several strategies have been developed that focus on the immune response or gene therapy. First, genetically engineered mouse (GEM) models enable the analysis of bladder tumors in immune-competent mice. A number of GEM models of urothelial cell carcinoma (UCC) employ the uroplakin II (UPII) promoter of the mouse UPII gene. By placing the UPII promoter upstream to drive the urothelium-specific expression of the SV40-T antigen, the GEM develops UCCs that bear a strong resemblance to human UCCs [3]. However, cancer cells in GEM models are less heterogeneous than human bladder cancer cells due to their similar origins, and as a result, GEM cannot be used to test the efficacy of novel immunotherapeutic agents. Alternatively, syngeneic tumors can be established by implanting bladder tumor cells formed by exposure to carcinogens. All studies involving syngeneic models have used MBT-2, MB49 or AY-27 cells [4]. Such syngeneic models exhibit good tumor acceptance rates and human bladder cancer mimicry. However, after the bladder tumors are detected with non-invasive *in vivo* imaging, such as MRI or bioluminescence imaging, the mouse dies within two to three weeks due to azotemia [5]. This outcome makes it impossible to observe long-term tumor responses after intravesical immunotherapeutic treatments. Therefore, the development of a bladder tumor model in mice with an appropriate growth rate that reflects the slow long-term progression of the disease is required.

The *c-myc* gene, which is translated to the c-MYC protein, is amplified and/or up-regulated in many types of cancer cells, and it plays critical roles in their malignant transformation, proliferation, apoptosis and metastasis [6]. It is believed that c-MYC regulates the expression of 15% of all genes and promotes cell cycle progression [7]. In quiescent cells, c-MYC expression is relatively undetectable. However, mitogenic stimulation rapidly induces the production of *c-myc* mRNA and protein and drives cells to enter the G1 phase of the cell cycle. Representative genes enhanced by c-MYC include *cdk4* and *cyclin D2*, which are essential for cell cycle progression [8]. Therefore, c-MYC is a potential target for the control of cancer proliferation. In the present study, we explored whether the moderate siRNA-induced down-regulation of *c-myc* expression in MBT-2 cells decreases cell proliferation and tumor growth in an orthotopic model of bladder cancer. These results may help facilitate the establishment of an appropriate murine bladder tumor model.

## RESULTS

### Establishment of MBT-2 cells expressing luciferase and GFP reporters

**Figure 1:**
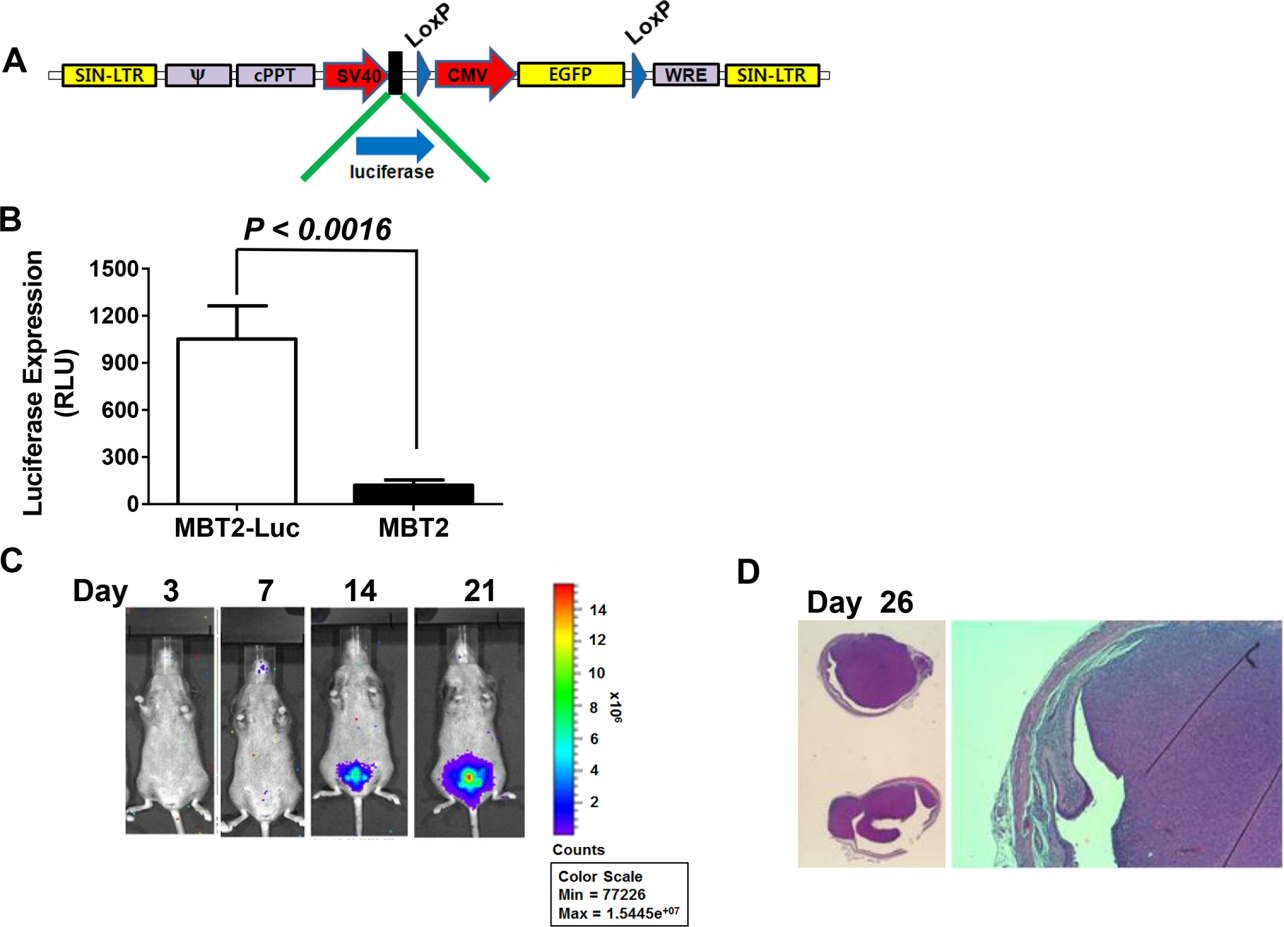
Construction of MBT2.Luc cells expressing the luciferase gene (**A**) The lentivirus shuttle vector pLoxLL3.7 was engineered to express a luciferase expression cassette under the SV40 promoter. Then, a four-plasmid-based lentiviral expression system was co-transfected with pMDLg/pRRE, pRSV-Rev and pCMV-G. (**B**) Approximately 1 × 10^5^ MBT2.Luc and MBT-2 cells were prepared in 12-well plates for 24 h. Cells were harvested for luminescence measurements as described in the Materials and Methods. The data represent the mean ± SD. (**C**) To establish the orthotopic bladder cancer model, 50 μl of 0.1 μg/mL poly-L-lysine was instilled for 15 minutes, and the bladder was voided. Approximately 2 × 10^6^ cells suspended in PBS were instilled intravesically via the urethra using a 22-gauge arterial puncture needle cannula. Luminescence images were captured at the indicated time points using INVIVO Lumina. (**D**) For histological analyses, all tumors from five mice were harvested at day 26. Tumor sections (5–10-mm thick) were affixed to slides, de-waxed with ethanol, and stained with hematoxylin and eosin (H&E).

MBT-2 cells are murine bladder cancer cells with epithelial characteristics. Their formation is induced by the administration of FANFT (N-[4-(5-nitro-2-furyl)-2-thiazolyl] formamide) to C3H/He mice [9]. In the past decade, significant advances have been made in the non-invasive evaluation of tumors within the body. Here, we induced MBT2.Luc cells to express luciferase and GFP to monitor tumor growth and the response to treatment using non-invasive *in vivo* imaging of the bladder. For this purpose, MBT-2 cells were transduced with lentiviruses that delivered both the luciferase and GFP genes (Figure 1A). MBT-2 cells that expressed GFP were isolated with a flow cytometer to construct MBT2.Luc cells. Luciferase gene expression in MBT-2.Luc cells was confirmed by luminescence. These MBT2.Luc cells highly expressed luciferase (Figure 1B) and were able to form a tumor in the bladder (Figure 1C). The bladder tumors grew quickly, filling more than 90% of the bladder's volume within 2 weeks (Figure 1D). This result suggested that the use of tumors implanted with MBT-2 and MBT2.Luc cells would not be appropriate to monitor the effects of intravesical treatment.

### Cell proliferation was inhibited by siRNA binding c-myc

**Figure 2:**
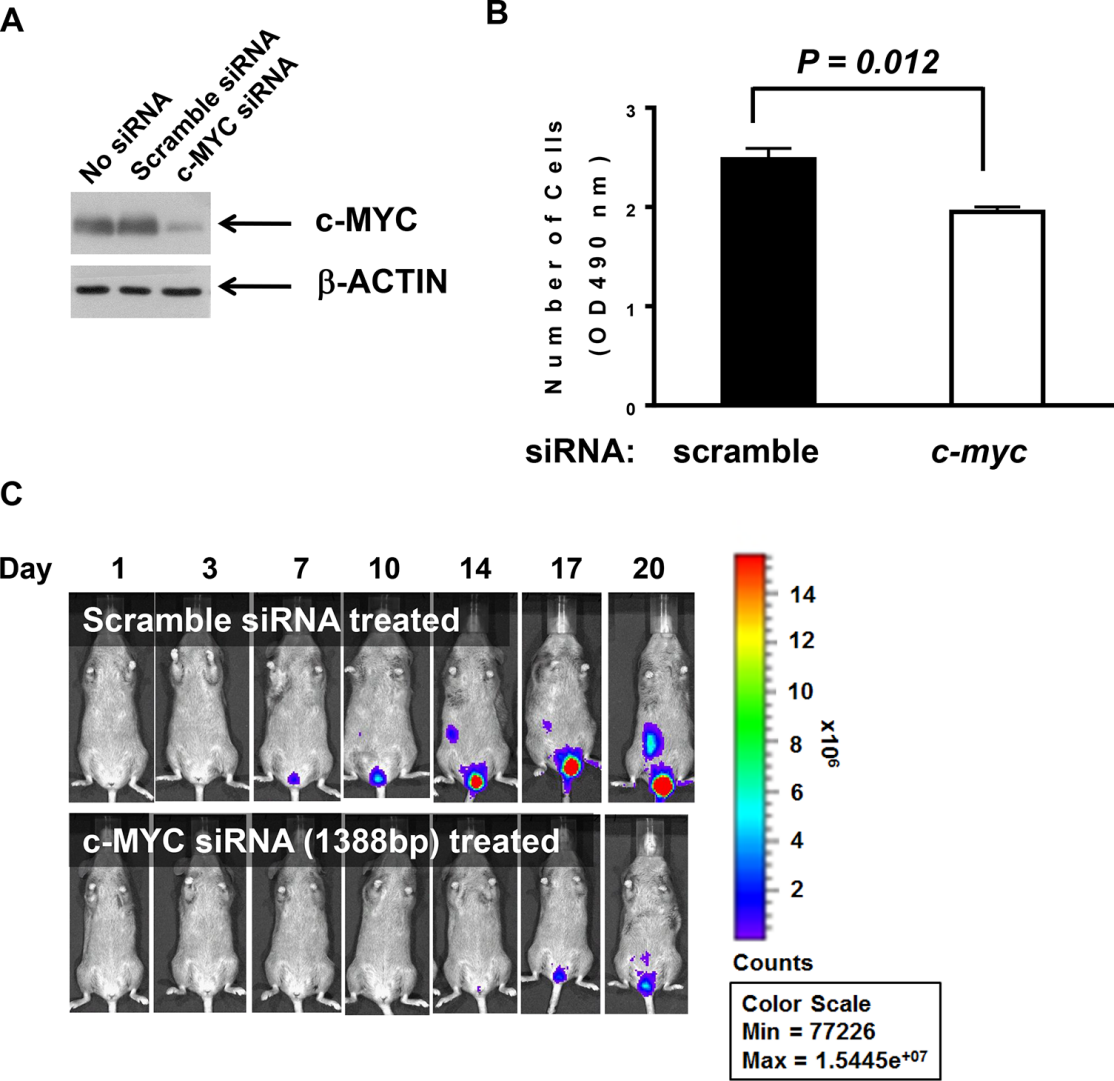
siRNA binding *c-myc* transcripts negatively influenced proliferation (**A**). MBT2.Luc cells were seeded at 2.5 × 10^5^ cells/well in 6-well plates to 40–50% confluence and transiently transfected with *c-myc* siRNA oligonucleotides or control scrambled siRNA. Then, cells were lysed with RIPA buffer. Approximately 30 mg of proteins were resolved on SDS-PAGE and transferred to PVDF for western blot analysis. Membranes were probed with antibodies against c-MYC and the subsequent corresponding secondary antibody was detected using the Enhanced Chemiluminescence (ECL) Plus kit. (**B**). Each cell line (3 × 10^3^ cells/well) was plated in a 96-well plate for 48 h and MTT assays were used to count viable cells. The data represent the mean ± SD. *P* values were determined by two-tailed paired *t*-tests. (**C**). MBT2.Luc cells (2 × 10^6^ cells/well) were plated in a 100-mm dish and transiently transfected with siRNA oligonucleotides binding mouse *c-myc* or scrambled siRNA. Approximately 1 × 10^6^ cells suspended in PBS were instilled in the bladder of a single mouse intravesically using a 22-gauge arterial puncture needle cannula. Luminescence images were taken at the indicated time points using INVIVO Lumina.

The transcription factor *c-myc* is up-regulated in many tumors [10–12] and plays critical roles in the progression of cancer cells [13]. In a previous study, we demonstrated that *c-myc* expression can be down-regulated to inhibit the *in vitro* cell proliferation and the *in vivo* tumor growth of bladder cancer cells [14]. Here, we examined whether siRNA against *c-myc* could effectively down-regulate *c-myc* expression and slow the proliferation of MBT-2 cells. Upon siRNA transfection, *c-myc* expression was suppressed, as confirmed by western blot analysis (Figure 2A). As expected, siRNA against *c-myc* significantly slowed cell proliferation rates (*P* = 0.012) relative to control siRNA (Figure 2B). Furthermore, we tested whether the reduced cell proliferation would translate to the *in vivo* tumor growth in the bladder. The tumor growth of MBT-2 cells transfected with siRNA against *c-myc* was slower than that of those treated with control siRNA (Figure 2C), which confirmed that *c-myc* controls the growth rate of MBT-2 tumors in the bladder.

### MBT-2 cells stably expressed shRNA against c-myc

**Figure 3:**
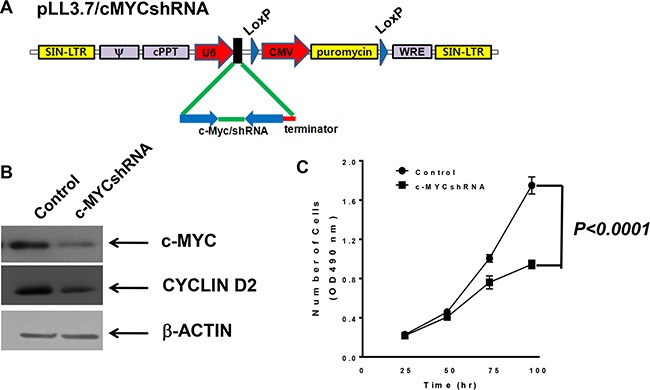
MBT2.cMYCshRNA cells expressing shRNA showed reduced numbers of *c-myc* transcripts (**A**) The lentivirus shuttle vector pLoxLL3.7 was engineered to express *c-myc* shRNA under the U6 promoter (pLL3.7/cMYCshRNA). Then, a four-plasmid-based lentiviral expression system was co-transfected with pMDLg/pRRE, pRSV-Rev and pCMV-G. (**B**) Cell lysates of MBT-2 and MBT2.cMYCshRNA were prepared and resolved on SDS-PAGE for western blotting. Western blotting using c-MYC-reactive or cyclin D2 antibodies showed the reduction of *c-myc* mRNA and the down-regulation of the cyclin D2 gene. (**C**) Approximately 3 × 10^3^ cells of each cell line were plated in a 96-well plate for 48 h, and proliferation was evaluated using MTT assays. *P* values were determined by two-tailed paired *t*-tests.

Next, MBT-2 cells were manipulated to reduce proliferation and consequently decelerate tumor growth. To construct MBT2.Luc cells, we utilized shRNA delivered by lentiviruses. Using a lentiviral vector system developed in a previous report [14], siRNA-inducing shRNA that bound to *c-myc* transcript was designed (Figure 2) and cloned into a modified pLoxLL3.7 vector (Figure 3A). Lentiviruses harboring c-MYCshRNA effectively silenced the expression of the *c-myc* gene (Figure 3B) and cyclin D2, which is controlled by *c-myc* transcription factor. These results demonstrated that transcribed shRNA was effectively converted to siRNA to silence *c-myc* expression. The stable MBT-2 cells expressing c-MYCshRNA were named MBT2.cMYCshRNA cells. To confirm that MBT2.cMYCshRNA showed reduced proliferation, cells were plated and evaluated compared with control shRNA-transfected MBT-2 cells. As shown in Figure 3C, MBT2.cMYCshRNA cells exhibited significantly slower proliferation rates (*P* < 0.0001).

### The *in vivo* growth rate was reduced with the down-regulation of c-myc expression

**Figure 4:**
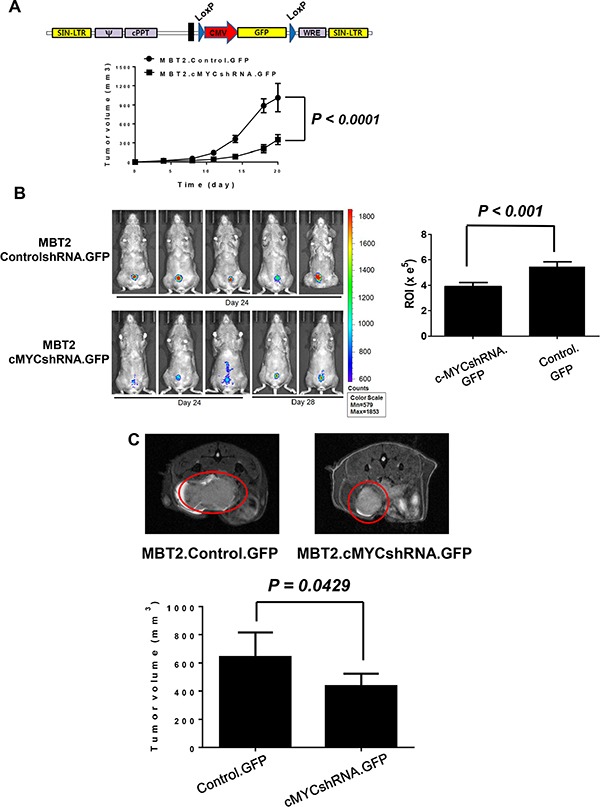
Repressed *in vivo* tumor growth of MBT2.cMYCshRNA.GFP in the mouse bladder (**A**) The lentivirus shuttle vector pLoxLL3.7 was engineered to express the GFP gene under the CMV promoter. Then, cells were infected with lentiviruses expressing GFP and fluorescence-expressing cells were sorted with a flow cytometer. Then, 1 × 10^6^ MBT2.cMYCshRNA.GFP or MBT2.ControlshRNA.GFP cells were subcutaneously implanted in five C3H/He mice. The tumor size was measured at the indicated time points. (**B**) Approximately 1 × 10^6^ cells suspended in PBS were instilled into the bladder intravesically using a 22-gauge arterial puncture needle cannula. Fluorescence images were taken at the indicated time points using INVIVO Lumina, and the ROIs of luminescence surrounding the tumor were calculated. *P* values were determined by two-tailed paired *t*-tests. (C) MRI images were acquired with a 7 T BioSpec spectrometer (Bruker, Germany). Tumor volumes were calculated for comparison between MBT2.cMYCshRNA.GFP and MBT2.ControlshRNA.GFP cells.

**Figure 5:**
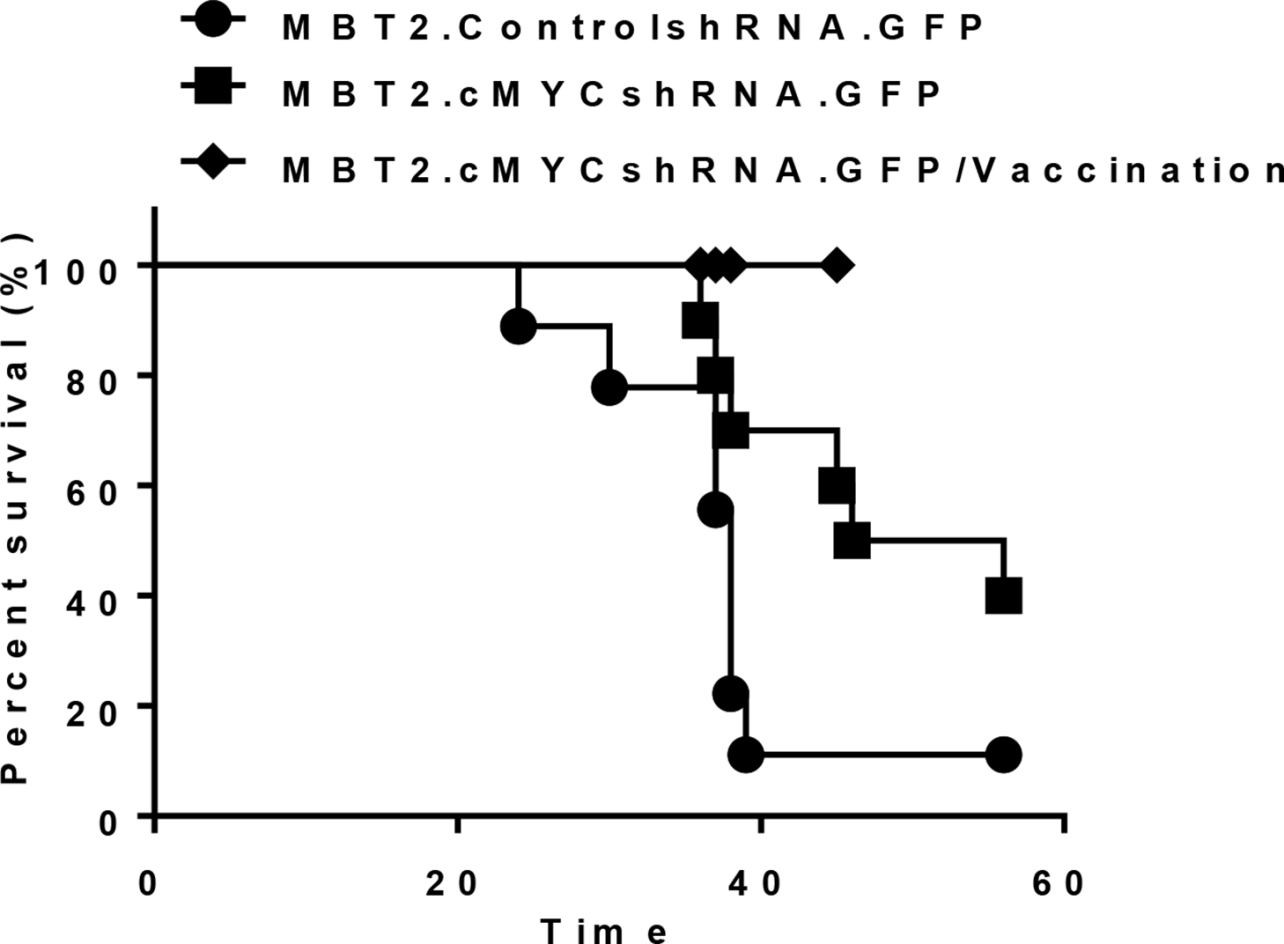
Prolonged survival of mice with orthotopic tumors in the bladder via the reduction of *c-myc* expression Intravesical xenografts in the bladders of mice were established using MBT2.cMYCshRNA.GFP (■) or MBT2.ControlshRNA.GFP (●) cells. The long-term survival rate was determined (*P* value, log-rank test). Furthermore, eight mice were included right after two subcutaneous vaccinations with 1 × 10^5^ of MBT-2 cells that were pre-treated with 10 Gy irradiation (♦)

To verify tumor formation and the control of the growth rate in the bladder, MBT2.cMYCcshRNA cells were infected with lentiviruses expressing GFP. The resultant cells were named MBT2.cMYCcshRNA.GFP, and their growth was monitored by fluorescence (Figure 4A). Before exploring the growth of orthotopic xenografts, the tumors of C3H/He mice were subcutaneously inoculated with MBT2.cMYCcshRNA.GFP cells and monitored relative to MBT2.ControlshRNA.GFP. As shown in Figure 4A, tumors implanted with MBT2.cMYCcshRNA.GFP grew significantly more slowly than those expressing ControlshRNA (*P* < 0.0001). Similar to the subcutaneous tumors, the syngeneic tumors in the bladder were smaller upon the reduction of *c-myc* expression with shRNA (Figure 4B, right) (*P* < 0.001). The region of intensity (ROI) calculations confirmed the lower growth rate of tumors composed of MBT2.cMYCcshRNA.GFP cells (Figure 4B, left). These results were verified by MRI analysis with statistical analysis (*P* = 0.0429), which was employed to visualize the exact size of tumors in the bladder (Figure 4C). The mice expressing either ControlshRNA or cMYCshRNA survived longer when inoculated with MBT2cMYCshRNA.GFP with statistical significance (*P* = 0.0462) (Figure 5). Furthermore, we subcutaneously vaccinated mice with irradiated MBT-2 cells twice and challenged them with MBT2cMYCshRNA.GFP cells. Upon vaccination, the survival significantly increased with no deaths at 45 days. These results suggest that MBT2.cMYCcshRNA.GFP cells represent a more appropriate murine model for the evaluation of immunotherapeutic treatments.

## DISCUSSION

The efficacy of drug therapies in human cancer patients cannot be reliably assessed with *in vitro* studies. Animal models are essential to the study of the efficacy of antineoplastic drugs. To effectively evaluate the effect of intravesical therapy in bladder cancer, an animal model must meet several requirements: 1) the tumor is of urothelial origin, with different stages of disease progression; 2) the tumor grows intravesically and can be directly exposed to antineoplastic drugs; and 3) it mimics the clinical course of human urinary bladder cancer. Three types of animal models have been proposed in previous reports: a chemically induced model, an orthotopic transplantable xenograft model, and a genetically engineered model [15]. However, most models are not suitable to evaluate therapy for NMIBC. The induction of primary bladder tumors with carcinogens requires several months, and tissues other than the urothelium can be unintentionally transformed [16]. Therefore, a transplantable orthotopic bladder tumor model is more useful for testing new anticancer therapies. Human bladder cancer cell lines can be introduced into the bladders of immunocompromised mice. However, tumors are not reliably acquired with cell lines other than KU7 [17], which are actually HeLa cells [18]. To improve tumor uptake, tumor cells can be directly injected into the bladder wall with or without laparotomy [19]. However, in this model, the tumor's epicenter is located in a muscle layer and grows expansively away from the lumen of the bladder; this growth pattern differs from the pattern of bladder cancer growth in humans, in which tumors originate from the urothelial surface of the bladder and grow intravesically. Moreover, this model is not suitable to evaluate the effect of the intravesical instillation of a novel drug to treat NMIBC because in this model, tumors invade muscle and are covered with normal urothelium, which disturbs the intravesical treatments that must be delivered directly to the tumor. Several studies have utilized orthotopic xenografts of human bladder cancer cells to explore drug efficacy.

Immunotherapy has been an effective treatment for bladder cancer since BCG therapy was introduced 40 years ago [1]. This successful immunotherapy appears to involve various immune cells, such as CD4^+^ and CD8^+^ lymphocytes, natural killer cells, granulocytes, macrophages, and dendritic cells [20]. Approximately 30% to 40% of patients do not respond to this type of therapy [21] due to an inadequate immune response. Recently, antibody-based immunotherapy has emerged as a promising option for bladder cancer. Of the twelve known monoclonal antibodies for cancer, programmed cell death (PD) inhibitors, such as atezolizumab (Roche/Genentech), nivolumab (Bristol-Myers Squibb) and pembrolizumab (Merck), are particularly effective in immunotherapy for bladder cancer. A number of potent immunotherapies, including adoptive cell therapy, vaccines and antibodies, are being investigated worldwide; however, no appropriate immune-competent animal model is currently available. Although a transplantable MBT-2 orthotopic model in C3H/HeJ mice has been widely used in BCG-based studies, syngeneic MBT-2 tumors of the bladder grow so rapidly that it is impossible for the immune system to mobilize, which occurs in human bladder cancer patients. However, if the expression of the *c-myc* gene is down-regulated by shRNA, MBT-2 cells may be employed in the development of successful immunotherapies.

Transplantable syngeneic orthotopic bladder tumor models present two major drawbacks. These models exhibit a low rate of tumor uptake, and the disruption of the urothelium by electrocautery or chemical applications to facilitate tumor uptake produces particularly invasive and aggressive tumors. Weldon and Soloway [22] reported that tumor cell implantation occurred in 13% of untreated mice, while the administration of *N*-methyl-*N*-nitrosourea before tumor introduction resulted in a tumor uptake rate of approximately 60%. Several modifications have been proposed to improve the success rate of orthotopic bladder tumor implantation. Shapiro *et al*. [23] reported a tumor uptake rate of approximately 60% with electrocauterization-induced mucosal injury before tumor introduction. In this modification, intravesical tumors were reduced by 24%. Pretreatment with HCl/KOH resulted in a tumor uptake rate of approximately 80 to 90%, of which approximately 20% were extravesical tumors [24]. In an HCl pretreatment model, the entire bladder wall is exposed to acid, which leads to extensive mucosal injury. Chan *et* *al*. [25] reported a high mortality rate and extensive bladder wall inflammation with HCl pretreatment. Thus, this model is too aggressive to be used to study local treatment effects. MBT-2 tumors grow rapidly in the bladders of mice. The mean survival time of animals in the orthotopic bladder cancer model is approximately 20–40 days. Thus, the rate of tumor development in the bladder and the animals’ survival time must be controlled. In this study, we present a novel orthotopic bladder cancer model in C3H/He mice in which the c-Myc expression of MBT-2 murine bladder cancer cells was inhibited to down-regulate the growth rate. However, *c-myc* is essential for p53-induced apoptosis in response to chemotherapeutic agent(s), so that chemotherapy acting through a mechanism inducing p53 expression is not efficacious in MBT2.cMYCshRNA.GFP cells stably down-regulating c-MYC proteins [26].

A number of reports address the role of the proto-oncogene *c-myc* in bladder tumors. Immunohistochemical staining with anti-c-MYC monoclonal antibodies revealed that the high expression of *c-myc* is associated with poorly differentiated bladder tumors [27]. With respect to copy number gains and amplifications in bladder cancer patients, alterations of the c-MYC gene, including copy number gains and amplifications, are linked to genetically unstable bladder cancers characterized by a high histologic grade and/or invasive growth [28, 29]. Therefore, this study inhibited the *c-myc* gene to reduce the rate cell proliferation and employed shRNA to down-regulate *c-myc* expression. Furthermore, shRNA delivered by lentiviruses decreased the number of *c-myc* transcripts rather than completely eliminating them. Conclusively, the novel orthotopic bladder cancer model in C3H/He mice described here modified the *c-myc* expression level of MBT-2 murine bladder cancer cells to control their growth rate. This model is ideal for the evaluation of novel intravesical immunotherapies.

## MATERIALS AND METHODS

### c-myc siRNA transfection

MBT-2 cells from a mouse bladder cancer cell line were cultured in RPMI 1640, 10% FBS and 1% penicillin/streptomycin. Approximately 2 × 10^5^ MBT-2 cells were prepared in a 6-well plate one day before transfection. Cells cultured to 60–80% confluence in a 6-well plate were transfected with Lipofectamine 2000 (Invitrogen, Carlsbad, CA, USA) following the manufacturer's protocol. We used the following siRNA: 5′-AAA GAG CAA GAA GAU GAG GAA-3′ and 5′-UUC CUC AUC UUC UUG CUC UUU TT-3′ for *c-myc* (Sigma-Aldrich); 5′-CAG UCG CGU UUG CGA CUG GTT-3′ and 5′-CCA GUC GCA AAC GCG ACU GTT-3′ as scrambled siRNA (Sigma-Aldrich, St. Louis, MO, USA).

### Western blot analysis

For western blot analysis, cells were lysed in RIPA buffer containing 10 mmol/L NaF and 5 mmol/L VO4 supplemented with protease inhibitors (Sigma-Aldrich). Approximately 30 μg of protein from the soluble fraction was resolved on SDS-PAGE and transferred to a PVDF membrane. Anti-cMYC (Cell Signaling, CA) or anti-b-actin (Sigma-Aldrich) antibodies were added to the membranes and subsequently reacted with peroxidase-conjugated polyclonal antibody (Sigma-Aldrich). Antibodies were detected using an Enhanced Chemiluminescence Plus kit (GE Healthcare, Pittsburgh, PA, USA).

### Construction and transduction of lentiviruses

We established stable MBT2.Luc cells by infecting lentiviruses harboring a luciferase gene under the SV40 promoter. A luciferase gene digested with *Nhe*I/*Hpa*I pGL3 control vector (Promega, Wisconsin, WI, USA) was cloned into *Xba*I/*Hpa*I sites of the lentiviral vector pLoxLenti3.7 (Addgene, Cambridge, MA, USA), which produced pLL3.7/Luc. Its expression was under the control of the SV40 promoter. Lentiviral particles produced from the pLL3.7/Luc vector were generated by co-transfection with three plasmids, pMDLg/pRRE, pRSV-Rev and pCMV-G, using Lipofectamine 2000 (Invitrogen). MBT2.Luc cells were infected with lentiviruses expressing luciferase, and GFP-positive MBT2.Luc cells were sorted with a flow cytometer. To reduce *c-myc* expression, two oligonucleotides encoding *c-myc*-specific shRNA were designed and cloned into *Apa*I/*Xba*I sites of the modified lentiviral vector pLoxLenti3.7, which produced pLL3.7/cMYCshRNA. The modified pLoxLenti3.7 vector exhibited the puromycin gene instead of GFP at pLoxLenti3.7. We selected the lentiviral vector that coded the *c-myc*-specific shRNA by cutting and sequencing the strands with restriction enzymes. Next, lentiviral particles were generated with the same procedures employed for the production of the pLL3.7/Luc lentivirus. MBT-2 cells transduced with lentivirus pLL3.7/cMYCshRNA were selected in the presence of 100 mg/mL puromycin. Next, pLL3.7/cMYCshRNA.GFP was constructed. MBT2.cMYCshRNA cells were infected with lentiviruses from the pLoxLenti3.7 vector, which produced MBT2.cMYCshRNA cells that expressed GFP.

### Cell proliferation assay

Approximately 3 × 10^3^ cells were plated in 96-well plates, and their levels of proliferation were measured at the indicated time points using the Cell Counting Kit-8 (Dojindo Laboratories, Rockville, MD, USA). All experiments were performed in triplicate.

### Animal studies

All animal experiments in this study were performed under specific pathogen-free conditions in accordance with the Guidelines for the Care and Use of Laboratory Animals of the National Cancer Center of the Republic of Korea. All animal experiments included five or six mice per group. To establish orthotopic bladder cancer models, eight-week-old female C3H/He mice were anesthetized with 1.75% isoflurane. A superficial 6–0 silk purse-string suture was placed around the urethral meatus before a lubricated 24 G angio-catheter was passed through the urethra into the bladder. For the pre-treatment, 50 μL of 0.1 μg/ml poly-L-lysine (Sigma-Aldrich) was instilled for 15 minutes, and the bladder was voided. A suspension of 2 × 10^6^ MBT2.Luc cells in 50 μL of PBS was instilled into the bladder, and the purse-string suture was tied down for 2 h. The presence of tumors in the bladder was confirmed by luminescence analysis. The same procedures were used in MBT2.cMYCshRNA cells.

### MRI experiments

For the *in vivo* MRI studies, the animals were anesthetized with isoflurane. To visualize the tumor lesions, rapid MRI examinations were performed, which involved the T2-weighted rapid acquisition of 12 axial slices of the bladder with the relaxation enhancement (RARE) sequence. Acquisition parameters were as follows: repetition time (TR) = 2500 ms, echo time (TE) = 35 ms, 256*256 matrix, field of view (FOV) = 2*2 cm, slice thickness = 0.7 mm, and RARE factor = 8. All MRI images were acquired with a 7 T BioSpec spectrometer (Bruker, Germany).
